# Bioengineered System for High Throughput Screening of Kv1 Ion Channel Blockers

**DOI:** 10.3390/bioengineering8110187

**Published:** 2021-11-16

**Authors:** George V. Sharonov, Oksana V. Nekrasova, Ksenia S. Kudryashova, Mikhail P. Kirpichnikov, Alexey V. Feofanov

**Affiliations:** 1 Shemyakin-Ovchinnikov Institute of Bioorganic Chemistry RAS, 16/10 Miklukho-Maklaya, 117997 Moscow, Russia; okatja@yandex.ru (O.V.N.); rekamoskva@mail.ru (K.S.K.); kirpichnikov@inbox.ru (M.P.K.); avfeofanov@yandex.ru (A.V.F.); 2 Department of Molecular Technologies, Institute of Translational Medicine, Pirogov Russian National Research Medical University, 1 Ostrovityanova, 117997 Moscow, Russia; 3 Institute of Experimental Oncology and Biomedicine, Privolzhsky Research Medical University, 10/1 Minin and Pozharsky Sq., 603950 Nizhny Novgorod, Russia; 4 Biological Faculty, Lomonosov Moscow State University, Leninskie Gory 1, 119992 Moscow, Russia

**Keywords:** potassium channel, channel blockers, spheroplasts, high throughput screening, agitoxin, charybdotoxin, kaliotoxin

## Abstract

Screening drug candidates for their affinity and selectivity for a certain binding site is a crucial step in developing targeted therapy. Here, we created a screening assay for receptor binding that can be easily scaled up and automated for the high throughput screening of Kv channel blockers. It is based on the expression of the KcsA-Kv1 hybrid channel tagged with a fluorescent protein in the *E. coli* membrane. In order to make this channel accessible for the soluble compounds, *E. coli* were transformed into spheroplasts by disruption of the cellular peptidoglycan envelope. The assay was evaluated using a hybrid KcsA-Kv1.3 potassium channel tagged with a red fluorescent protein (TagRFP). The binding of Kv1.3 channel blockers was measured by flow cytometry either by using their fluorescent conjugates or by determining the ability of unconjugated compounds to displace fluorescently labeled blockers with a known affinity. A fraction of the occupied receptor was calculated with a dedicated pipeline available as a Jupyter notebook. Measured binding constants for agitoxin-2, charybdotoxin and kaliotoxin were in firm agreement with the earlier published data. By using a mid-range flow cytometer with manual sample handling, we measured and analyzed up to ten titration curves (eight data points each) in one day. Finally, we considered possibilities for multiplexing, scaling and automation of the assay.

## 1. Introduction

Despite the progress in precision medicine such as genetic-, cell-based- and immunotherapies, synthetic and peptide drugs are still indispensable and most effective for various pathologies. The accelerated appearance of protein structure data and new drug-discovery algorithms gives rise to vast amounts of new drug candidates. This stimulates the demand for fast, affordable and reliable techniques for first-line drug screening [1].

Target proteins used in screening assays can be expressed in *E. coli* or in eukaryotic cells. The former has a much higher yield and cost efficiency; however, this is challenging when membrane proteins are needed [2,3]. The latter requires time- and reagent-consuming handling and provides limited control of membrane insertion and accessibility of the target protein. For eukaryotic cells, ligand binding and affinity may be also affected by their lateral interactions with other proteins and complex allosteric regulations [4,5,6]. The expression of transmembrane proteins in the membrane of *E. coli* might be a superior combination with advantages of high *E. coli* expression and avoidance of eukaryotic cell-related difficulties. However, the membranes and translation machinery of *E. coli* are not suited for the folding and embedding of mammalian transmembrane proteins. For these reasons, using receptor chimeras has been proposed, in which the membrane-facing portion is endogenous for *E. coli*, while ligand-binding sites facing the extracellular space are replaced by the corresponding target receptor domains [7,8,9].

Such chimera of the prokaryotic potassium channel KcsA (KcsA-Kv1.3), with the voltage-gated potassium channel Kv1.3, made it possible to effectively reconstitute the physiological tetrameric structure of the ion-selective pore of Kv1.3 [8]. Using the pET system (Novagen), the expression level of KcsA-Kv1.3 in the *E. coli* inner membrane was estimated to be as high as 10^5^ receptors per cell [10]. This chimeric receptor was readily accessible for ligands after the disruption of the outer bacterial wall, and the obtained spheroplasts were used for the evaluation of ligand binding. Complex formation between chimeric receptors and their ligands occurred directly on the outer surface of the plasma membrane, thus allowing us to omit the labor-intensive purification and functional reconstitution of the chimeric membrane protein. We have demonstrated the utility of this system for measuring Kv1.3 ligand affinities and for searching for new potential blockers within complex mixtures such as animal venom [11].

In our previous studies, we used confocal laser scanning microscopy (CLSM) to analyze the binding of rhodamine-labeled peptide blocker agitoxin 2 (R-AgTx2) to hybrid KcsA-Kv1.3 channels expressed in the membrane of *E. coli* spheroplasts [10,11,12,13]. R-AgTx2 was used as a fluorescent probe in a competitive binding assay to determine the affinity of non-labeled peptides and small organic molecules to the target channel. Despite several advantages, this detection method was the most time-consuming and laborious part of the analysis. It required: bulk and expensive CLSM equipment; precise control of a concentration of *E. coli* cells in the reaction mixture, which was placed in a 12-well silicon chamber attached to a glass slide pretreated with poly-L-lysine; immobilization of cells on the glass by centrifugation of the whole chamber using special centrifuge adaptors; precise adjustment of the focal plane; manual data acquisition and quantitative analysis of each spheroplast; and the subtraction or minimization of the background due to adsorption of a fluorescent ligand on the glass (that also unpredictably decreases the concentration of the available ligand). At the same time, flow cytometry is the gold standard for quantitative and statistical analysis of micrometer-sized fluorescent particles. However, there are still no conventional pipelines for its use in ligand-binding screening assays.

**Figure 1:**
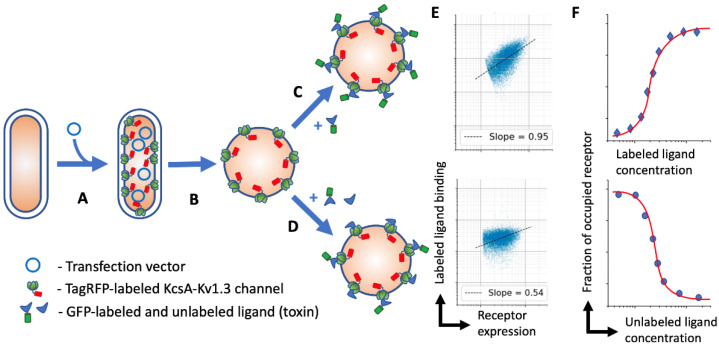
Schematic representation of the assay pipeline. (**A**) *E. coli* are transfected with an expression vector coding KcsA-Kv1.3-RFP chimeric channel; (**B**) on the day of the experiment, the *E. coli* envelope is disrupted with a lysozyme/EDTA solution to make the channel accessible for water-soluble compounds; (**C**,**D**) spheroplasts are incubated with either a labeled ligand or its mixture with an unlabeled (same or other) ligand; (**E**) spheroplasts are analyzed by flow cytometry and the amount of fluorescent ligand binding per unit of expressed receptor (fraction of occupied receptor) is derived. (**F**) The data on the fraction of occupied receptors is fitted with appropriate binding curves and dissociation constants are calculated.

In this study, we evolved our spheroplast-based assay for use with flow cytometry and created an automated data analysis protocol. We constructed the KcsA-Kv1.3 hybrid, tagged with a red fluorescent protein (TagRFP), to discriminate the receptor expressing spheroplast from the cell debris and system noise and normalize ligand binding by the amount of target receptor on each spheroplast (Figure 1). This assay allowed us to acquire and analyze about ten ligand titration datasets on a single midrange flow cytometer within one day. The developed assay was easily transferable and possessed high throughput that could be further increased by combining several receptors (tagged with different fluorescent proteins) in one reaction volume, as well as by using 96-well plate loaders that are commonly available nowadays.

## 2. Materials and Methods

### 2.1. Recombinant Toxins

Recombinant peptide toxins agitoxin 2 (AgTx2), charybdotoxin (ChTx) and kaliotoxin 1 (KTx) were produced, as described earlier [12], using expression plasmids pET23d-MalE-L1-AgTx2, pET23d-MalE-L1-ChTx and pET23d-MalE-L1-KTx. Peptide toxins AgTx2 and ChTx, both tagged N-terminally with eGFP were used as fluorescent probes in the binding studies. Design and production of AgTx2-GFP, in which AgTx2 and eGFP moieties were separated by a 45-aa linker L1, was described earlier [14]. To obtain the analogous hybrid protein ChTx-GFP, a gene encoding AgTx2 in the previously obtained plasmid pET23d-GFP-L1-AgTx2 [14] was replaced by the ChTx coding sequence from pET23d-MalE-L1-ChTx, using KpnI/HindIII sites. Correct cloning of the recombinant gene in the resulting plasmid pET23d-GFP-L1-ChTx was confirmed by sequencing both strands (Evrogen, Russia).

Hybrid protein ChTx-GFP was expressed and purified as described earlier [14]. Briefly, *E. coli* Rosetta-gami (DE3) pLysS cells were transformed with a plasmid pET23-GFP-L1-ChTx and cultivated at 37 °C in Terrific Broth (TB) supplied with 100 mg/L ampicillin, 15 mg/L kanamycin, 12.5 mg/L tetracycline and 34 mg/L chloramphenicol. Protein expression was induced with 0.1 mM isopropyl-D-thiogalactopyranoside (IPTG) and cells were further grown at 20 °C for 22 h. After sonication of *E. coli* biomass, ChTx-GFP was purified from the lysate by affinity chromatography on a Ni-NTA Sepharose Fast Flow column (GE Healthcare) following the manufacturer’s protocol. The protein was desalted using a PD-10 column (GE Healthcare) in PBS (pH 7.4) and stored at +4 °C in the dark in the presence of 0.02% sodium azide for two months. Concentration of ChTx-GFP was determined by absorption spectroscopy using molar extinction coefficient for eGFP (ε_489_ = 55,000 M^−1^ cm^−1^).

### 2.2. Cloning of KcsA-Kv1.3-RFP

A gene coding for KcsA-Kv1.3 hybrid protein [8] was previously cloned into the NcoI/HindIII sites of pET-28a expression vector (Novagen) [10]. The tag coding sequence 6xHis was located at the 3′-terminus of *KcsA-Kv1.3* gene between BglII and HindIII sites, and TAA stop-codon overlapped HindIII recognition sequence. To obtain KcsA-Kv1.3 C-terminally tagged with TagRFP, BglII site was changed for BamHI site for the convenience of subsequent cloning into pET-28a. For this, *KcsA-Kv1.3* gene was amplified by PCR using oligonucleotide primers K-f1 (5′-TTCTCCATGGGCCCACCCATGCTGTCCGGT; NcoI site is underlined) and K-r1 (5′-TTCTGGATCCGCGGTTGTCGTCGAGCATTCGCTC; BamHI site is underlined) to get DNA Fragment I. Then, a gene encoding TagRFP was amplified by PCR from the plasmid pTagRFP-C (Evrogen, Russia) using oligonucleotide primers R-f1 (5′-TTCTGGATCCGGATCAACTAGTGGTTCTGGTATGGTGTCTAAGGGCGAAGA; BamHI site and ATG start codon are underlined) and R-r1 (5′-TTCTCTCGAGATTAAGTTTGTGCCCCAGTTTGCTA; XhoI site is underlined) to get DNA Fragment II, in which 5′-terminal 27 aa coded for the linker sequence GSGSTSGSG, which separated KcsA-Kv1.3 and TagRFP in the hybrid protein. Two PCR fragments, namely Fragment I and Fragment II after digestion with restriction endonucleases NcoI/BamHI and BamHI/XhoI, respectively, were ligated with NcoI/XhoI-digested pET-28a vector. Resulting expression plasmid pET28a-KcsA-Kv1.3-RFP coded for the hybrid KcsA-Kv1.3-RFP with C-terminal 6xHis tag. Sequence analysis of the recombinant gene was performed for both strands (Evrogen, Russia).

### 2.3. Spheroplast Preparation

Spheroplasts were prepared according to the previously developed protocol [10]. Briefly, *E. coli* cells (BL21 (DE3) strain) transformed with pET28a-KcsA-Kv1.3-RFP plasmid were cultivated in a minimal M9 medium at 37 °C up to OD_560_ = 0.8, then induced with 50 μM IPTG and further grown for 18 h. Cells were harvested by centrifugation (5000× *g*, 10 min, 4 °C), incubated in buffer A (10 mM Tris–HCl, pH 8.0, 0.5 m sucrose) containing lysozyme (20 μg/mL) for 7 min on ice, then diluted twofold with buffer B (10 mM Tris–HCl, pH 8.0, 0.3 mM ethylenediamine tetraacetic acid) and further incubated for 20 min. Then MgCl_2_ was added to a final concentration of 10 mM to stabilize the spheroplasts. Obtained in this way, the spheroplast suspension has OD_560_ of about 0.5 and was diluted for binding assay as described further.

### 2.4. Flow Cytometry

For ligand binding analysis, a series of ligand dilutions were prepared in 12 × 75 mm flow cytometry test tubes. For this, 45 µL of toxin solution with a ten times higher concentration than the maximal concentration on titration curve was prepared. This solution was serially diluted with 3.16-fold step (13.9 µL of toxin solution + 30 µL buffer). These toxin dilutions were supplemented with 270 µL of spheroplast suspension at 10^6^ cells/mL to give 300 µL final reaction mixtures. For analysis of displacement of GFP-labeled toxins by unlabeled toxins, spheroplast suspensions were supplemented with 1.1× concentration of GFP-labeled toxin before mixing with unlabeled toxin dilutions. All dilutions were made in a binding buffer: 0.25 M sucrose, 0.3 M EDTA, 2 mM KCl, 50 mM NaCl, 10 mM MgCl_2_, 0.2% BSA and 50 mM Tris–HCl, pH 7.5.

**Figure 2:**
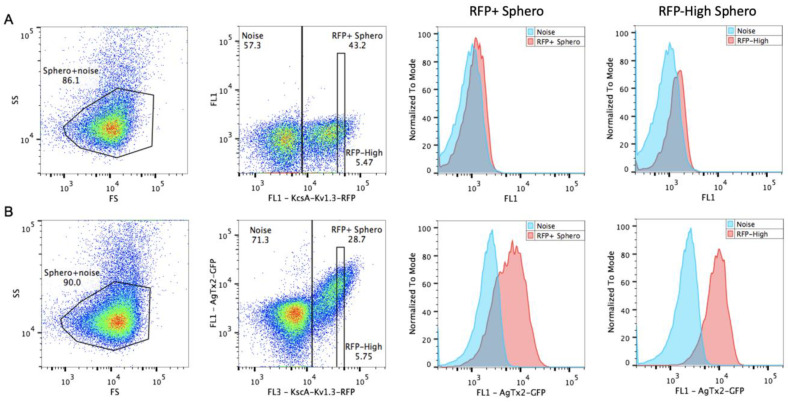
Analysis of ligand binding to spheroplasts by conventional flow cytometry approaches. Cells before (**A**) and after (**B**) incubation with AgTx2-GFP are shown. The first column represents gating on spheroplasts based on light scattering. Spheroplast regions are indicated. The second column shows dot plots of spheroplasts according to RFP and GFP fluorescence. All spheroplasts are RFP-positive, while RFP-negative events are debris and system noise. Gates for all RFP-positive events (RFP+) and events within the narrow RFP range (RFP-High) are presented. Histograms of spheroplast distribution according to the AgTx2-GPF bindings (red), compared to the noise (blue) in the same sample, are presented for RFP+ and RFP-High spheroplasts, respectively.

As revealed earlier, the binding equilibrium in our system is reached after 30 min of incubation [11]. To ensure equilibrium conditions, we analyzed samples after 90–120 min of incubation without removing toxins. Data acquisition was carried out with FC-500 flow cytometer (Beckman Coulter, Brea, CA, USA) using 488 nm excitation for both GFP and RFP and fluorescence detection with FL1 (505–545 nm) and FL3 (610–630 nm) channels for GFP and RFP, respectively. To minimize background fluorescence, samples were run at low speed that gave approx. 100–500 events/s. Events were triggered by side scatting (SSC) so that RFP-labeled cells constituted approx. 30% of events (Figure 2). Remaining events were also present in the samples of filtered binding buffer without cells, so were likely to be related to system noise (either electronic or optical) but not to the dim spheroplasts. Triggering by FL3 did not give better results, since fluorescent cells were either partially lost or constituted less than half of the total events.

### 2.5. Flow Cytometry Data Analysis

Automated data analysis was performed by a dedicated script after pre-processing with commercial software. The script was available in user-friendly Jupyter notebook format where data were processed stepwise, and user input-output was realized through an internet browser. This script is designed to process multiple datasets at once and can be easily adjusted for someone’s actual needs.

Initial preprocessing was done with used FlowJo v10 software (Treestar Inc., Wilmington, DE, USA). This was used for gating on cells by forward and side scatter, followed by gating of RFP-positive events (Figure 1) and exporting of gated data in FCS format. This step was preferable since it allowed one to convert data into a conventional FCS format (our flow cytometer import data in LMD file format) and to filter out off-target events such as system noise, cell debris, clumped and untransfected cells (without Kv1.3-RFP in our case). We also created a script for this preprocessing but found it more complicated and less robust than commercial software.

Filtered data were processed stepwise by a dedicated script, written in the Python programming language and available as a Jupyter notebook for running either on a local machine or in a kaggle data cloud (https://www.kaggle.com/georgesharonov/fcsgroupfit, accessed and executed on 14 November 2021). Data processing workflow was designed for high throughput analysis by processing all similar data in a single script execution. The processing steps were detailed within the notebook. Two types of data were supported: (i) titration curves of labeled ligand binding and (ii) curves of labeled ligand displacement by unlabeled ones. All data of a single type could be processed simultaneously. At the first step, data entries were associated with certain conditions and concentrations according to specific strings entered by the user and found within data filenames. Then gated data presented as ligand binding (LB) versus receptor expression (RE) level were fitted with linear function: LB = background + slope × RE. Slope values were calculated and represented as amount of ligand bound per unit of receptor. These slope values for ligand binding or ligand displacement were fitted with appropriate curves in order to calculate ligand-receptor dissociation constants (K_d_). For binding of labeled ligands, K_d_ represented a ligand concentration that produced half-maximal binding. For displacement curves, concentrations of unlabeled ligands that provided displacement of 50% of the labeled ligand (IC_50_) were derived by curve-fitting and used to calculate an apparent dissociation constant of unlabeled ligands (K_ap_) with Cheng-Prusoff equation: K_ap_ = IC_50_/(1 + [L]/K_d_), where [L] and K_d_ were a concentration and a dissociation constant of the labeled ligand, respectively.

To estimate fitting accuracy, we calculated standard deviation of K_d_ values from covariance matrixes for fitted and actual data. For benchmarking of the different data processing pipelines, we used single datasets for a straight titration of the labeled ligand. SD values, in this case, represented deviations of the data in these single titrations. For displacement titration, we measured displacement curves and calculated K_ap_ in duplicate or triplicate. SD for K_ap_ represented a deviation in these replicates.

## 3. Results

### 3.1. Optimization of Spheroplast Detection by Flow Cytometry

Flow cytometry is one of the most suitable methods for the quantitative and statistical analysis of 1–100 µm particles. Thus, we aimed to adapt our spheroplast-based ligand-binding assay for use with flow cytometry. Measurement of 1 µm particles that are near to the lower size limit is challenging since the scattering (commonly used to trigger measurements when a particle is in interrogation point) of such particles is relatively low. First, we tried to detect spheroplasts with light scattering. The best results were obtained when triggering was done by a side scatter. This resulted in an approximately two-fold increase in the event count for the spheroplast containing the sample, as compared to the buffer alone. Spheroplast appeared on a scattering dot plot at the same place as the system noise that prevented discrimination of the noise and spheroplasts by scattering. In this case, spheroplasts still can be discriminated by their labeling with a fluorescent ligand, but this does not work at a low ligand binding because of the overlap of a labeled spheroplast with the noise. Such conditions are crucial for building proper binding curves and calculating binding constants. This limitation may be overcome by labeling spheroplasts with a separate dye that allows one to discriminate them from off-target events.

For better spheroplast discrimination, we evaluated several vital dyes. Good results were obtained with the BCECF-AM fluorescein-based dye (1 µM for 30 min RT), but this was not suitable to use with the GFP-labeled ligands. The DRAQ5 DNA stain and FM1-43 membrane stain were analyzed as possible alternatives. Despite DRAQ5 (10 µM for 1 h) being excited and detected with nearly optimal wavelengths (633 nm and 735–775 nm, respectively), it gave only a minor shift of labeled cells relative to non-target events and did not allow their discrimination. FM1-43 (5 µM for 10 min) provided bright staining of spheroplasts and enabled their discrimination from noise but had a large fluorescence spillover into the FL1 channel designated for GFP-tagged ligands. We failed to compensate for this spillover adequately probably because the apparent FM1-43 spectrum represents an overlay of its different spectra in different membrane structures.

Another approach that we used to discriminate target receptor-expressing spheroplasts was their tagging with a fluorescent protein (FP). This could be either (i) another FP-coding plasmid in addition to the target receptor-bearing plasmid; (ii) expression of unconjugated FP from the same plasmid; or (iii) chimera of a target receptor with FP. We took advantage of the third variant because it provided equal expression of target receptor and FP and allowed one to quantify the receptor amount in each cell [15]. We tagged KcsA-Kv1.3 with TagRFP (KcsA-Kv1.3-RFP) and produced clones expressing this chimera. Even with suboptimal 488 nm excitation, RFP gave a sufficiently good resolution of receptor-expressing cells from unwanted events (Figure 2). In addition, RFP had no spillover into the FL1 (GFP) channel (Figure 2A). Ligand binding to spheroplasts was linearly proportional to the KcsA-Kv1.3-RFP expression, resulting in a prolonged and inclined population (Figure 2B). In order to retrieve the receptor occupancy ratio from this data, we have evaluated several approaches.

### 3.2. Optimization of Flow Cytometry Data Processing

We have evaluated several approaches of flow cytometry data processing and quantification. Within each approach, we measured binding curves that were fitted with a one-site binding equation. In order to compare these approaches, they were evaluated both qualitatively and quantitatively by analyzing the curve shape, the comparison of the coefficient of determination (R^2^) and the consistency with the published data. Two sets of binding data were used for this comparison: the binding of AgTx2-GFP and ChTx-GFP.

First, we used a conventional approach to calculate the mean or median values for the RFP-positive population minus that of the RFP-negative population. In this case, ligand binding did not reach saturation at high concentrations, both for AgTx2-GFP and ChTx-GFP, but instead increased linearly at concentrations above 10 nM (data not shown). Although the resulting *R*^2^ values were rather high (0.95 and 0.97 for AgTx2-GFP and ChTx-GFP), the lack of saturation with up to 100 nM was contradictory to previous results and revealed some general inconsistency of this approach. As a result, calculated K_d_ values appeared to be much higher than they should be (6 and 32 nM for AgTx2-GFP and ChTx-GFP, respectively). To fix this inconsistency, we noticed that the intensity of the ligands bound to RFP-positive cells had a large deviation along the GFP-axis (log scale CV for 3.2 nM AgTx2-GFP is 75%) that could cause an inadequate binding curve shape. Therefore, we tried to decrease it by analyzing the binding for cells within a narrow range of RFP intensity (Figure 2, RFP-High region). By narrowing the analyzed population, its deviation decreased less than twice (for 3.2 nM AgTx2-GFP CV decreased to 45%) and gave no improvement of the binding curve shape.

**Figure 3:**
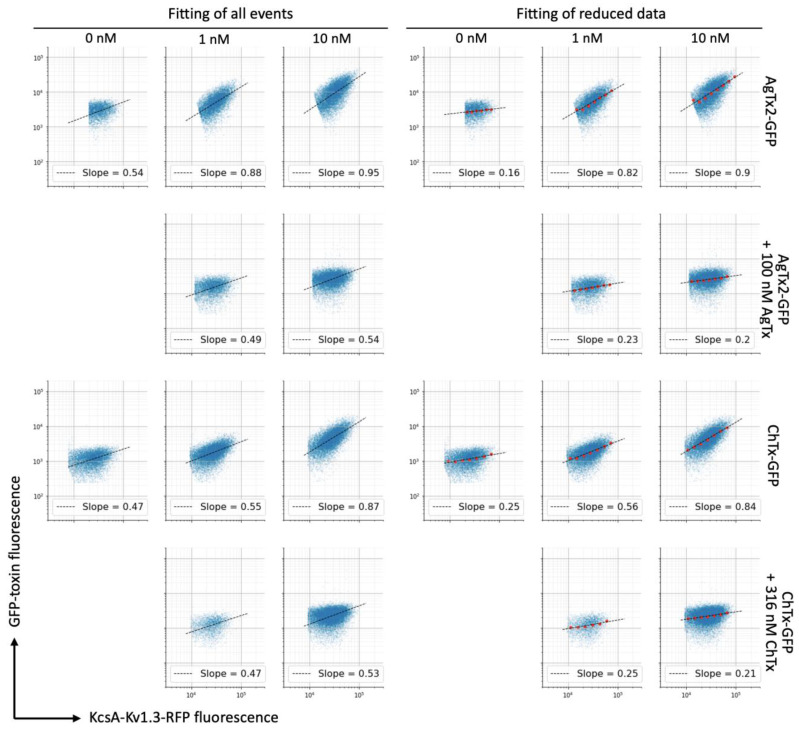
Analysis of a fraction of an occupied receptor from flow cytometry data. Two strategies are presented: fitting of all cells (**left** panel) and fitting or reduced data (**right** panel). Reduced data are calculated as a maximum of normal cell distribution along the FL1-GFP axis within the narrow window of RFP intensity and presented as red dots. Fitting was done with linear functions. Slopes of the fitted lines (that are proportional to the occupied receptor fraction) are presented on each plot. In the first and third rows, direct binding of AgTx2-GFP and ChTx-GFP at indicated concentrations are presented. The second and fourth rows show nonspecific binding of labeled toxins at the excess of unlabeled counterparts.

Since the conventional analysis of flow cytometry data did not provide satisfactory results, we evaluated other approaches. We took advantage of the KcsA-Kv1.3-RFP quantity measurement in each cell. In the absence of cooperative interactions, the amount of ligand bound per cell was linearly proportional to the amount of receptor that was reachable by the ligand. Indeed, on a dot plot of GFP-toxin fluorescence vs. RFP-receptor fluorescence, the RFP-positive population appeared inclined and elongated, reflecting a linear relationship between receptor expression and ligand binding (Figure 2). This also indicated that all the KcsA-Kv1.3-RFP that was expressed in a cell was available for toxin binding. The slope of this linear relationship corresponded to the fraction of occupied receptors. Initially, we determined this slope by linear fitting all RFP-positive events (Figure 3). Calculated in such a way, binding data were fitted with a one-site binding curve that provided an affordable curve shape and adequate K_d_ values of 0.2 ± 0.04 nM and 0.86 ± 0.19 nM and *R*^2^ of 0.983 and 0.977 for AgTx2-GFP and ChTx-GFP, respectively.

**Figure 4:**
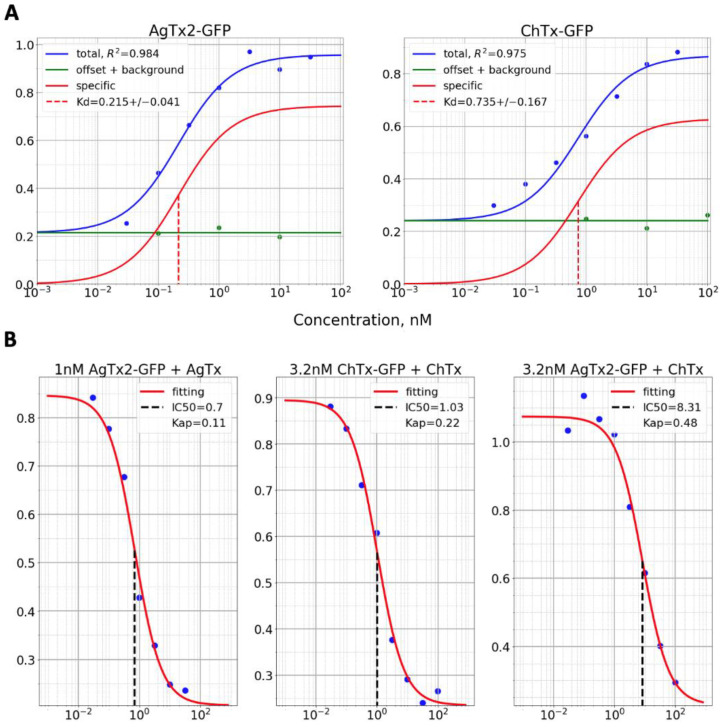
Analysis of ligand binding curves for direct (**A**) and displacement (**B**) titrations. (**A**) Experimental data points and fitted binding curves for AgTx2-GFP (**left**) and ChTx-GFP (**right**) are presented in blue. Nonspecific binding data and curves are presented in green. Binding curves with subtracted nonspecific binding are shown in red. Values for *R^2^* and calculated K_d_s are indicated on the graphs. Concentrations corresponding to K_d_ are also shown as vertical dashed lines. (**B**) Displacement of 1 nM AgTx2-GFP (first graph), 3.2 nM ChTx-GFP (second graph) or 3.2 nM AgTx2 (third graph) with unlabeled AgTx2 (first graph) or ChTx (second and third graph). Experimental data points and fitted curves are depicted. The concentration of unlabeled toxins that displace 50% of the labeled ligands are shown as vertical dashed lines and indicated by numbers. Derived K_ap_ values are also presented.

Although the results were consistent, this approach has potential pitfalls. It is not uncommon for flow cytometry that cells/events are clustered near to some prevailing values with only a few cells distant enough to draw the relationship between two parameters of interest. The approximation is especially sensitive to such clustering. In order to avoid the disturbance of the approximation curve by a nonuniformity of event distribution and to increase the robustness of data processing, we propose using data reduction along one of the axes. In this case, data are binned along the *x*-axis and all data corresponding to a certain (binned) x-value are reduced to a point (or few points) along the *y*-axis. Such processing gives eventually distributed x values with corresponding single (or few but an equal amount of) y values, thus removing the effects of event clustering. Multiple approaches can be used to reduce data along the *y*-axis. It can be a substituted with mean or median value, or it can be fitted with a certain statistical distribution or a mixture of these distributions. A superposition of two Gaussian distributions, for example, has been used by us to discriminate between responsive and unresponsive cells [16]. Here, we reduced data by the substitution of scattered along *y*-axis data (GFP-Toxin) with the maximum of its normal distribution. Reduced data were fitted with lines to obtain slope values that, in turn, were fitted with a one-site binding curve (Figure 3 and Figure 4). K_d_ values in this case were found to be 0.2 ± 0.04 nM and 0.74 ± 0.17 nM, and *R*^2^ − 0.984 and 0.975 for AgTx2-GFP and ChTx-GFP, respectively (Figure 4, Table 1). Although *R*^2^ values are slightly lower than without data reduction, this approach is beneficial in certain cases, such as the one mentioned above [16]. Here, the second approach with data reduction also appeared to be suited better for our data especially for calculation of background with the excess of unlabeled toxin (Figure 3).

### 3.3. Measurement of Dissociation Constants of Labeled and Unlabeled Ligands

Our approach was evaluated using several Kv1.3 ligands with known dissociation constants. These are unlabeled recombinant channel blockers from scorpion venom, AgTx2, ChTx, KTx and labeled GFP-tagged chimeras AgTx-GFP and ChTx-GFP. First, we calculated K_d_ values by analysing the AgTx-GFP and ChTx-GFP binding curves with the Kv1.3-RFP expressing spheroplasts, which were found to be 0.20 ± 0.04 nM (Mean ± SD) and 0.74 ± 0.17 nM, respectively (Figure 3, Table 1). This is close to the values reported earlier: 0.21 nM for AgTx2-GFP in our system and 1.8–2.6 nM for ChTx (Table 1). Notably, in the presence of an excess of unlabeled ligand (100 nM of AgTx2 or 316 nM of ChTx), the binding of labeled ligands is completely inhibited (Figure 4). This indicates that there is no nonspecific binding in our system at the concentration of labeled ligands of up to 100 nM, which is supported by the well-defined saturation of binding curves at high ligand concentrations.

Although labeled ligands allow direct evaluation of binding, they are not readily available but are prepared through sophisticated procedures of construction, cloning, purification and labeling. This compromises the throughput of the screening procedure and complicates the search of Kv binding substances within complex and/or unknown mixtures. Furthermore, labeling may affect the activity and/or binding properties of a ligand, thus any data obtained with a labeled ligand is not valid for unlabeled ligands in general. We have used a common approach for the analysis of unlabeled ligands by their ability to displace labeled ones with known K_d_. In our experiments, we used both AgTx2-GFP and ChTx-GFP with the constants determined above and unlabeled toxins AgTx2, ChTx and KTx. We used 1–3.2 nM of labeled toxins, which is approx. 4–5 times higher than expected K_d_ values of unlabeled ligands. The use of higher concentrations of labeled ligands provides several advantages: it increases the range of labeled ligand concentrations to be used, thereby diminishing possible artifacts of using very diluted samples; it decreases the effect of the displaced ligand on its concentration in the media; it speeds up equilibration; and it increases the effective signal (RFP-positive population slope) and allows better curve resolution in the low signal range. Disadvantages of high concentrations, namely, nonspecific binding, high background and high ligand consumption, are negligible in our case if concentrations of labeled toxins are below 10 nM.

The K_ap_ values for AgTx2, ChTx and KTx that were obtained by analyzing the AgTx2-GFP or ChTx-GFP displacement are presented in the Table 1. Binding constants of AgTx2 and ChTx were 2–4 times lower than that of their labeled variants. These results are expected since toxin (M_r_ = 4–5 kDa) labeling with a large fluorescent protein (M_r_ = 26 kDa) is likely to compromise its binding. AgTx2 and ChTx have different binding sites on the Kv1.3 channel pore [17]. Thus, we have measured the apparent dissociation constant of ChTx by its ability to displace either ChTx-GFP or AgTx2-GFP. ChTx was found to be more effective in displacing ChTx-GFP than AgTx2-GFP, as indicated by twice lower K_ap_ in the ChTx-GFP displacement experiment. This confirms that the overlap of binding sites of two competitive ligands does affect K_ap_, but even if this overlap is moderate, our assay gives K_ap_ values that are the same order of magnitude compared to real K_d_.

**Table 1:** K_d_ values obtained by direct titration of GFP-toxins or by their displacement with unlabeled ones and their comparison to our previous and literature data.

Toxin	Dissociation Constants, nM *			K_d_ or IC_50_, nM
	Direct Titration (GFP-Toxins)	Apparent, by Displacement of Toxin Indicated	Our Previous Data	Literature Data
AgTx2	0.20 ± 0.04	0.10 ± 0.02 (AgTx2-GFP), *n* = 2	0.21	0.004 [18], 0.2 [19], 6.4 [8]
ChTx	0.74 ± 0.17	0.20 ± 0.02 (ChTx-GFP), *n* = 2 0.4 ± 0.1 (AgTx2-GFP), *n* = 2	-	1.8 [20], 2.6 [21]
KTx		0.04 ± 0.02 (AgTx2-GFP), *n* = 3	0.78	0.02 [22], 0.65 [21], 0.1–2 [23]

* Data are presented as Mean ± SD.

## 4. Discussion

Developed assays have several advantages over commonly used alternatives such as radioligand assay, surface plasmon resonance, fluorimetry and microscopy. First, they allow cells to be analyzed without wash that better preserves equilibrium conditions required for the correct estimation of K_d_. This is especially crucial for ligands with micromolar binding constants that usually have a lifetime of bound ligand-receptor complex in the order of a second [24]. Secondly, flow cytometry has a substantial ability for quantitative fluorescence detection, with a broad dynamic range that has been around for four decades for most instruments and up to six decades with modern instruments and appropriate dyes [25,26]. Thirdly, it integrates a signal for single cells that, on the one hand, provides sufficient signal intensity and, on the other hand, allows one to take into account the expression differences between cells. Lastly but not the least, flow cytometry has reach possibilities for multicolor analysis and assay multiplexing yet requires fluorescent ligands.

Two genetically encoded fluorescent ligands, namely, AgTx2-GFP and ChTx-GFP, were used to bind assays as fluorescent probes. As shown by us earlier [14,27], chimeric constructs (FP-Tx) consisting of fluorescent proteins (FP), namely, eGFP or TagRFP, fused with potassium channel toxins from scorpion venom (Tx) largely retain a high affinity of the natural toxins for the target Kv1 channels. These FP-Tx ligands have distinct advantages over fluorescent peptide blockers, which are obtained by the chemical conjugation of a peptide toxin with a fluorophore. FP-Tx proteins are expressed in *E. coli* and purified from bacterial biomass with a high yield of about 100 mg per 1 L of bacterial culture; their purification procedure is rather simple and does not require any renaturation step. Introduced by means of bioengineering techniques of a fluorescent tag in a predetermined position (either N- or C-terminal) of the peptide toxin results in a more definite assessment of the binding activity of a fluorescent peptide [14].

In order to estimate the possibilities and limitations of the assay, the molecular details of blocker interaction with a natural and chimeric channel should be considered. The common feature of the peptide blocker binding is an occlusion of the pore with the side chain of a lysine residue, which is accompanied by the formation of multiple bonds between the peptide and amino acid residues surrounding the pore [28,29]. Since the pore occlusion is the essential feature of any Kv1-channel blocker interacting with a channel at the extracellular side, the binding sites of peptide blockers and small organic molecule blockers overlap. As a result, fluorescently labeled peptide blockers can be used in competitive binding assays to recognize non-labeled blockers among both peptides and small organic molecules. For the hybrid Kv1-KcsA channels expressed in the *E. coli* membrane, this was demonstrated using laser scanning confocal microscopy as a detection method and tetraethylammonium as a competitive ligand [11].

Many other residues besides lysine are involved in the interaction of peptide blockers with the Kv1 channel. For example, 16 residues of AgTx2 interact with 11 residues of KcsA-Kv1.3, according to a molecular modeling study [29]. Thus, while a secondary structure of peptide blockers from scorpion venoms, such as AgTx2, ChTx and KTx, is similar, their differences in amino acid composition change a peptide-channel interaction pattern in a complex way and define the observed variations in affinity (Table 1).

Notably, a number of bioactive small molecules (such as 4-aminopiridine, clofazimine and psoralenic compounds) inhibit Kv1 channels from the inside of the cell [17], thus preventing implementation of a competitive binding assay using Kv1-KcsA hybrids. Consequently, our flow cytometry approach can be used to screen unlabeled small organic blockers for their binding with the external lining of the pore. If necessary, FP-peptide blockers can be substituted in the presented assay by small fluorescently labeled ligands, and their affinities can be estimated by direct titration, using the approach described above for FP-peptide ligands.

Multiplexing capabilities available with flow cytometry are highly demanded in pharmacological screening. Modern cytometers allow an analysis of up to 8–16 fluorophores simultaneously, with 4–5 colors being common practice [30]. The most obvious way to use this capability is to mix cells expressing different channels/receptors tagged with various FPs. The use of conventional FPs and flow cytometers allows one to unmix signals of four FPs (CFP, YFP, DsRed and TagFP635) along with the GFP-labeled ligand [31]. This panel can be expanded with novel near-infrared FPs [32]. Alternatively, a substantial increase in multiplexing can be achieved using fusions with two or more spectrally distinct FPs with adjusted brightness [15,33]. This was used to discriminate up to 20 tags with flow cytometry and has further extension potential [33].

Multiplexing will allow the screening of ligands for binding to several channels/receptors simultaneously with identical conditions (sample preparation, concentrations, reaction timing, etc.). This is a common task in the design of targeted drugs that bind to a certain receptor with minimal affinity to homologs. For example, targeting the Kv8.1 subunit ought to affect only brain tissue with minimal effect on other tissues [34], while selective targeting Kv1.3 is promising in the treatment of autoimmune disorders since it is one of the major Kv channels (along with Kv1.5 [35]) expressed in leukocytes and was shown to be associated with rheumatoid arthritis and type 1 diabetes mellitus [28]. Nonselective blockers that bind several multiplexed channels can be used in displacement experiments with unlabeled drug candidates. If differences in K_d_s for these channels is within one order of magnitude it can be accounted for during analysis while larger differences will require a set of labeled ligands for different (groups of) channels.

## 5. Conclusions

Expressed in spheroplasts, KcsA-Kv1 chimeras have been proved to be useful and reliable tools to study channel blockers. They allow one to measure channel blocker binding with the outer lining of the channel. In the absence of the intracellular and membrane-facing parts of the eukaryotic channel, they provide specific sites for drug targeting and more precise affinity measures due to the absence of complex allosteric regulation of the channel and off-target drug activities. Here, we adapted this expression system for use in flow cytometric binding assays, where the KcsA-Kv1 expression level was taken into account, and which provides much better quantification, multiplexing and scaling capabilities compared to the microscopy setups used earlier. The developed assay has everything required for use in large-scale automated screenings of drug candidates targeting the Kv1 channel pore.

## Data Availability

Data used in the current work is available as two datasets for straight and displacement titrations: https://www.kaggle.com/georgesharonov/kv13-straight-titration (accessed on 14 November 2021). https://www.kaggle.com/georgesharonov/kv13-displacement-titration (accessed on 14 November 2021). Analysis pipeline is deposed as Jupyter notebook: https://www.kaggle.com/georgesharonov/fcsgroupfit (accessed on 14 November 2021).
